# Structural Elucidation of a Novel Lipooligosaccharide from the Cold-Adapted Bacterium OMVs Producer *Shewanella* sp. HM13

**DOI:** 10.3390/md17010034

**Published:** 2019-01-08

**Authors:** Angela Casillo, Rossella Di Guida, Sara Carillo, Chen Chen, Kouhei Kamasaka, Jun Kawamoto, Tatsuo Kurihara, Maria Michela Corsaro

**Affiliations:** 1 Department of Chemical Sciences, University of Naples “Federico II”, Complesso Universitario Monte S. Angelo, Via Cintia 4, 80126 Naples, Italy; ross.diguida@gmail.com; 2 Characterisation and Comparability Laboratory, National Institute for Bioprocessing Research and Training. Fosters Avenue, Mount Merrion. Blackrock, Co., A94 X099 Dublin, Ireland; sara.carillo@nibrt.ie; 3 Institute for Chemical Research, Kyoto University, Uji, Kyoto 611-0011, Japan; chenchen@mbc.kuicr.kyoto-u.ac.jp (C.C.); kama.kinoko144@mbc.kuicr.kyoto-u.ac.jp (K.K.); jun_k@mbc.kuicr.kyoto-u.ac.jp (J.K.); kurihara@scl.kyoto-u.ac.jp (T.K.)

**Keywords:** psychrophile, lipooligosaccharide (LOS), structure elucidation, NMR spectroscopy, outer membrane vesicles (OMVs), MALDI-TOF mass spectrometry, cold adaptation

## Abstract

*Shewanella* sp. HM13 is a cold-adapted Gram-negative bacterium isolated from the intestine of a horse mackerel. It produces a large amount of outer membrane vesicles (OMVs), which are particles released in the medium where the bacterium is cultured. This strain biosynthesizes a single major cargo protein in the OMVs, a fact that makes *Shewanella* sp. HM13 a good candidate for the production of extracellular recombinant proteins. Therefore, the structural characterization of the components of the vesicles, such as lipopolysaccharides, takes on a fundamental role for understanding the mechanism of biogenesis of the OMVs and their applications. The aim of this study was to investigate the structure of the oligosaccharide (OS) isolated from *Shewanella* sp. HM13 cells as the first step for a comparison with that from the vesicles. The lipooligosaccharide (LOS) was isolated from dry cells, purified, and hydrolyzed by alkaline treatment. The obtained OS was analyzed completely, and the composition of fatty acids was obtained by chemical methods. In particular, the OS was investigated in detail by ^1^H and ^13^C NMR spectroscopy and MALDI-TOF mass spectrometry. The oligosaccharide was characterized by the presence of a residue of 8-amino-3,8-dideoxy-*manno*-oct-2-ulosonic acid (Kdo8N) and of a d,d-heptose, with both residues being identified in other oligosaccharides from *Shewanella* species.

## 1. Introduction

Cold-adapted bacteria are extremophiles that are able to thrive in permanently cold environments. Some of these habitats are exposed to temperatures below 5 °C [1], and for this reason cold-adapted microorganisms have developed unique physiological tools to survive in these harsh conditions. Cold habitats are also considered as surprising reservoirs of biotechnological molecules such as antibiofilm molecules [2], surfactants [3], cold-active enzymes [4], antifreeze proteins, glycoproteins, and polysaccharides [5,6,7]. Many psychrophiles have been reported to produce outer membrane vesicles (OMVs) [8]. It has been reported that the OMVs produced by a cold-adapted bacterium contain putative proteolytic enzymes, which can serve to degrade high molecular weight molecules present in the surrounding cells [9,10] helping the bacterium in the survival of such harsh conditions.

OMVs are small spherical particles commonly secreted from Gram-negative bacteria, and their role is not yet fully understood [11,12]. They are derived from the outer membrane (OM) by sticking out a portion of the envelope, and, for this reason, they usually contain phospholipids, outer membrane and periplasmic proteins, and lipopolysaccharides (LPSs). The latter molecules are the main components of Gram-negative bacterial OMs, for which they account for approximately 75% of the surface. LPSs are amphiphilic molecules with a conserved structure [13] fundamental for the viability and survival of Gram-negative bacteria and cyanobacteria [14,15,16], since they significantly affect the cell homeostasis due to their influence in cellular integrity and OM permeability [17].

Although LPS is also considered to be a major component of OMVs, very little is known about the LPS structure belonging to OMVs, which deeply limits the knowledge about their role in OMVs. This is mainly because the isolation and characterization of LPS from OMVs is usually impaired by the low amount of produced material. Thus, it is important to use a bacterial strain that abundantly produces OMVs to overcome this problem. Characterization of the structure of the LPS isolated from the bacterial cells, followed by a comparison with the LPS isolated from the OMVs, would greatly contribute to our understanding of the role of LPS in OMVs. Moreover, LPS structural characterization allows us to deepen the knowledge of psychrophilic marine Gram-negative bacteria and their mechanisms of adaptation to these prohibitive environments.

Herein, we describe the structural characterization of the carbohydrate backbone of the lipooligosaccharide (LOS) isolated from *Shewanella* sp. HM13, a cold-adapted Gram-negative bacterial strain that was isolated from the intestine of a horse mackerel and was found to produce a large amount of OMVs [18]. The OMVs produced by this strain carry a single major cargo protein named P49. This high relative abundance of a single cargo molecule in OMVs is a unique characteristic of this strain. The strain is thus expected to be useful as a host for extracellular production of recombinant proteins, including membrane proteins, as cargoes of OMVs by using the OMV-targeting mechanism of P49. Structural characterization of the molecules constituting the outer membrane of this strain is important for understanding of the mechanism of biogenesis of OMVs and their applications.

The fatty acids were removed from the lipooligosaccharide by mild hydrazinolysis (*O*-deacylation), followed by an alkaline treatment with 4 M KOH (*N*-deacylation). The obtained product was investigated by mono- and two-dimensional NMR spectroscopy, MALDI-TOF mass spectrometry, and chemical analysis.

## 2. Results and Discussion

### 2.1. LOS Extraction and Purification

**Figure 1:**
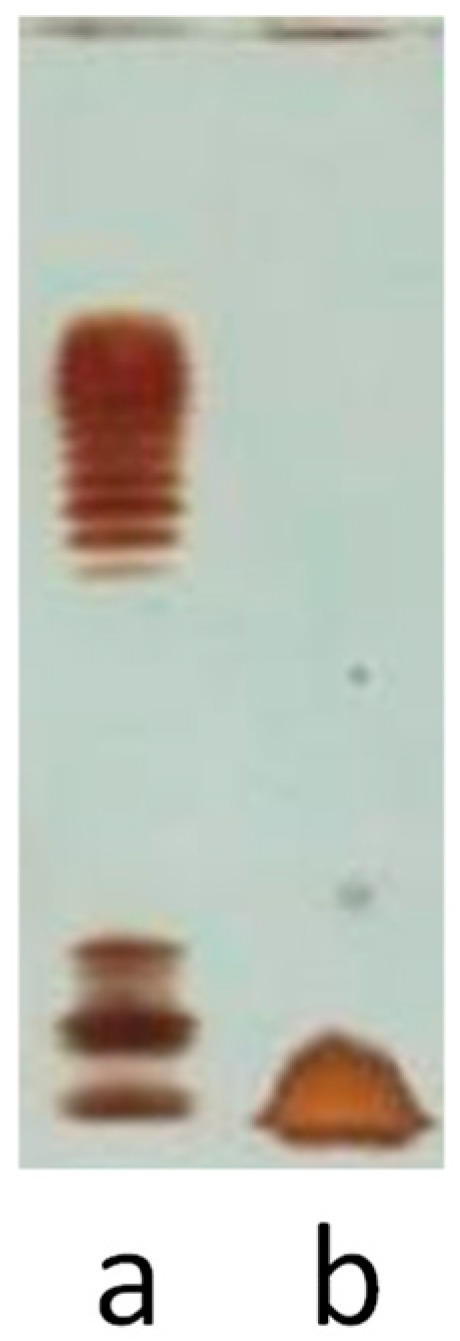
Analysis of the lipooligosaccharide (LOS) (Lane **b**) fraction from *Shewanella* sp. HM13 by 14% deoxycholate-polyacrylamide gel electrophoresis analysis (DOC-PAGE). The gel was stained with silver nitrate and the LOS was compared with the lipopolysaccharide (LPS) from *Escherichia coli* O127: B8 (Lane **a**).

*Shewanella* sp. HM13 cells were grown in Luria Bertani (LB) medium at 4 °C, as described in the Experimental section, and the LPS was isolated from dried cells using the phenol/chloroform/light petroleum (PCP) method [19], with a yield of 2.4%. As illustrated in Figure 1, sodium deoxycholate-polyacrylamide gel electrophoresis analysis (DOC-PAGE) showed, after silver nitrate gel staining, a fast migrating species typical of rough LPS (e.g., LOS). The cellular debris were also extracted by the phenol/water method [20], obtaining the same fast-migrating DOC-PAGE LOS together with proteins and nucleic acids (data not shown).

The compositional monosaccharides analysis of the obtained LOS revealed the presence of d-glucose (d-Glc), 2-amino-2-deoxy-d-glucose (d-GlcN), l-*glycero*-d-*manno*-heptose (l,d-Hep), and d-*glycero*-d-*manno*-heptose (d,d-Hep). Methylation analysis indicated the presence of terminal glucose, 2-substituted glucose, 2-substituted heptose, terminal heptose, and 2,6,7-trisubstituted heptose.

In addition, gas chromatography-mass spectrometry (GC-MS) analysis of fatty acid methyl esters showed the presence of C12:0(3OH), C13:0(3OH), C14:0(3OH), C12:0, C13:0, C14:0, and C15:0 as the major components.

### 2.2. LOS Deacylation

**Figure 2:**
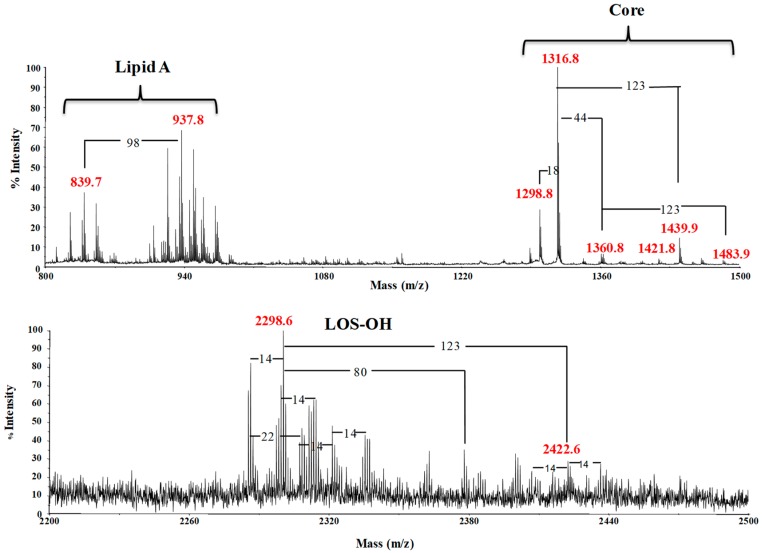
Negative ions MALDI-TOF mass spectrum recorded in reflectron mode of the *O*-deacylated LOS (LOS-OH). The upper spectrum displays the mass range 800-1500 *m/z*, whereas the lower one the mass range 2200-2500 *m/z*. Average masses are reported in the spectrum. The notation ‘14 Da’ indicates differences of single CH_2_ in the fatty acid chains. ‘18 Da’ indicates the loss of water. ‘44 Da’ indicates the loss of CO_2_. ‘80 Da’ indicates differences of phosphate groups. ‘98 Da’ indicates the loss of phosphate plus water. ‘123 Da’ indicates differences of phosphoethanolamine groups.

Alkaline degradation by mild hydrazinolysis of the LOS afforded an *O*-deacylated LOS, named LOS-OH, which was analyzed by negative ions MALDI-TOF (Figure 2). The spectrum revealed the presence of a cluster of ions, attributable to the LOS-OH molecule. At higher molecular masses, the signal at *m*/*z* 2298.6 was assigned the following composition: Hex_3_Hep_3_Kdo8NGlcN_2_P_3_[C13:0(3OH)][C14:0(3OH)] (Calculated [M−H]^−^ = 2298.84 Da), thus suggesting the presence of a residue of 8-amino-3,8-dideoxy-*manno*-oct-2-ulosonic acid (Kdo8N). The appearance of the Kdo8N monosaccharide was not surprising, since it has often been reported for *Shewanella* LPSs [21,22,23,24]. Differences of ±14 Da with respect to the main signal at *m*/*z* 2298.6 are attributable to the different lengths of fatty acids substituting the GlcN residues. A less intense signal was observed at *m*/*z* 2422.6, suggesting the presence of an additional phosphoethanolamine. Moreover, signals attributable to a core oligosaccharide and a lipid A, arising from an in-source β-elimination at the glycosidic bond between the Kdo8N and the lipid A, were also displayed [25]. The signals at *m*/*z* 1360.8 and 1483.9 were both attributed to the core fragments, with the difference of 123 Da being due to the additional phosphoethanolamine. The signals of the decarboxylated core fragments were clearly visible at *m*/*z* 1316.8 and 1439.9 [25]. Finally, further fragmentation with losses of 18 u could explain the signals at *m*/*z* 1298.8 and 1421.8. The LPS-OH was de-*N*-acylated by strong alkaline hydrolysis, and the obtained oligosaccharide, named **OS**, was submitted for full 2D NMR analysis.

### 2.3. NMR Spectroscopic Analysis of OS

2D NMR spectroscopy (^1^H,^1^H double quantum filtered-correlation spectroscopy (DQF-COSY), ^1^H,^1^H total correlation spectroscopy (TOCSY), ^1^H,^1^H rotating frame Overhauser enhancement spectroscopy (ROESY), ^1^H,^13^C distortionless enhancement by polarization transfer-heteronuclear single quantum coherence (DEPT-HSQC), and ^1^H,^13^C heteronuclear multiple bond correlation (HMBC) allowed for the assignment of all the proton and carbon chemical shifts of **OS**. The experiments indicated the pyranose rings for all the residues. Anomeric configurations were deduced by both proton and carbon anomeric chemical shifts, and by the ^1^*J*_C1,H1_ values obtained from the coupled ^1^H, ^13^C DEPT-HSQC experiment (Table 1).

**Figure 3:**
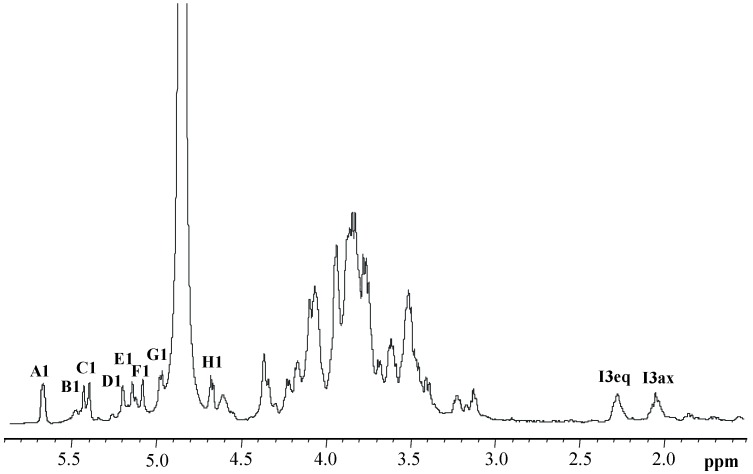
^1^H NMR spectrum of the oligosaccharide (**OS**) obtained by strong alkaline hydrolysis of the LOS isolated from *Shewanella* sp. HM13. The spectrum was recorded in D_2_O at 298 K at 600 MHz. The letters refer to the residues as described in Table 1.

**Figure 4:**
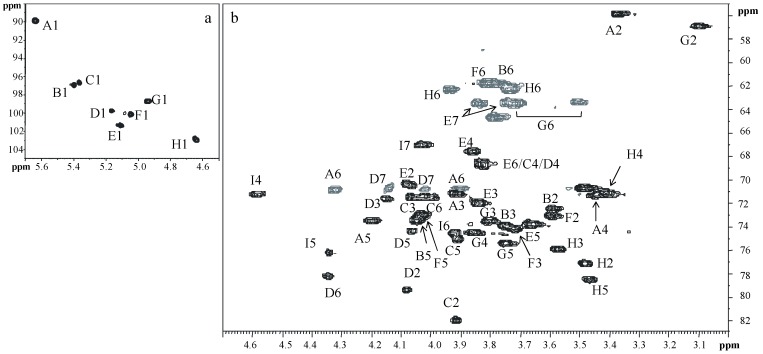
Anomeric (**a**) and carbinolic regions (**b**) of ^1^H-^13^C DEPT-HSQC spectrum of **OS** of the LOS from *Shewanella* sp. HM13. The spectrum was recorded in D_2_O at 298 K at 600 MHz. The letters refer to the residues as described in Table 1.

The ^1^H NMR spectrum of **OS** displayed eight main anomeric signals, indicating at least the equivalent number of monosaccharides, as shown in Figure 3 (residues **A**–**H**). In addition, two proton signals, at δ 2.01 and 2.25 ppm, confirmed the presence of a deoxy sugar (residue **I**). Starting from the chemical shift of H1 of residue **A**, the spin system in the COSY and TOCSY spectra revealed a *gluco* configuration, since it showed the typical ^3^*J*_H,H_ vicinal coupling constant values. This residue was identified as the phosphorylated vicinal glucosamine residue (α-GlcNI), due to the multiplicity of its anomeric signal (doublet of doublets, ^3^*J*_H1,H2_ = 3.6 Hz and ^3^*J*_H1,P_ = 7.0 Hz) and the chemical shift of its C2 (δ 55.8 ppm), as shown in Figure 4. Similarly, residue **G** was identified as the distal glucosamine (β-GlcNII) of the lipid A backbone. The ROESY and HMBC experiments (see below) indicated that residues **G** and **A** were linked through a linkage (1→6). Residues **C**, **D**, and **E** were recognized to be α-heptoses from their spin-system connectivities revealed in the COSY, TOCSY, and ROESY spectra. Indeed, in the TOCSY experiment, only the connectivities between H1 and H2 were clearly visible, indicating small values for the ^3^*J*_H1,H2_ and ^3^*J*_H2,H3_. Residue **C** was found to be 2-substituted, since the chemical shift of its C2 was shifted downfield to 82.1 ppm (the reference value for an unsubstituted residue is 71.9 ppm) [26].

Residue **E** did not show any downfield chemical shifts, and therefore was assigned to a terminal non-reducing α-heptose. Spin system **C** was identified as a 2,6,7-trisubstituted heptose, since its C2, C6, and C7 carbon chemical shifts occurred at 79.4, 78.2, and 70.8 ppm, respectively. The d,d-configuration for this residue was suggested based on the presence of this type of residue in other *Shewanella* LOSs, and from the strong similarities of the proton and carbon chemical shifts of this residue with those already reported [27]. The *gluco* configuration for the spin systems of residues **B**, **F**, and **H** was inferred from the typical ^3^*J*_H,H_ vicinal coupling constant values. By comparison with the ^13^C chemical shifts of unsubstituted residues [28], only one low-field shifted signal was identified for the C2 of residue **H**, indicating that **B** and **F** were terminal non-reducing residues. Finally, **I** was identified as a Kdo8N residue based on the characteristic diastereotopic proton signals at δ 2.01 and 2.25 ppm, and from the high-field chemical shift of its C8 signal at δ 44.8 ppm. The α configuration for this residue was inferred from the coupling constant values of ^3^*J*_H7,H8a_ (8 Hz) and of ^3^*J*_H7,H8b_ (3.2 Hz) [29].

**Figure 5:**
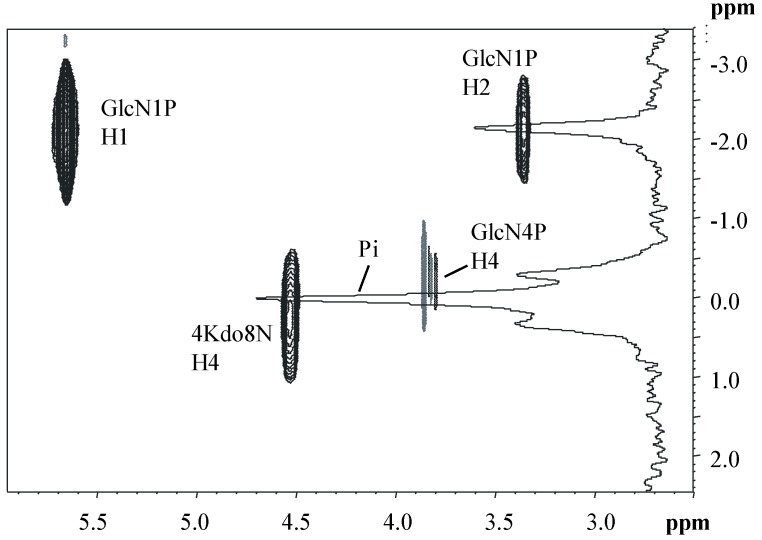
Expansion of the ^1^H-^31^P HSQC spectrum of the **OS** of the LOS from *Shewanella* sp. HM13. The spectrum was recorded in D_2_O at 298 K at 400 MHz.

The ^1^H-^31^P HSQC experiment of **OS** indicated three-bond correlations for the phosphorus signals, with ^1^H signals as follows: H1 and H2 of residue **A** with ^31^P signal at δ −2.1 ppm, H4 of **G** with ^31^P signal at δ −0.2 ppm, and H4 of **I** with ^31^P signal at δ 0.3 ppm, as shown in Figure 5. Therefore, based on these results and in agreement with other oligosaccharide structures from *Shewanella* species [23,24], it was possible to hypothesize that the additional phosphoethanolamine is linked through a phosphoanhydride linkage to the phosphate group at position O-4 of the Kdo8N.

**Figure 6:**
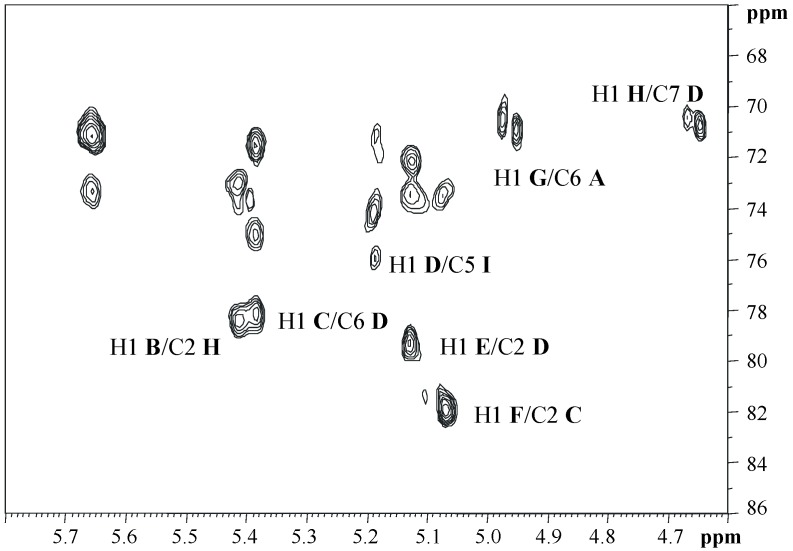
Anomeric region of the ^1^H,^13^C HMBC spectrum of the **OS** oligosaccharide. The spectrum was recorded in D_2_O at 298 K at 600 MHz. The letters refer to the residues as described in Table 1.

The sequence of the monosaccharides and the linkage positions were obtained from the ROESY and HMBC experiments. The latter spectrum, as illustrated in Figure 6, revealed the following inter-residue correlations: H1 of **G** with C6 of **A**, H1 of **D** with C5 of Kdo8N (residue **I**), H1 of **C** with C6 of **D**, H1 of **E** with C2 of **D**, H1 of **H** with C7 of **D**, H1 of **F** with C2 of **C**, and H1 of **B** with C2 of **H**. The ROESY spectrum confirmed this sequence, since it revealed the following dipolar couplings: H1 of **D** with H5 of **I**, H1 of **C** with H6 of **D**, H1 of **H** with both H7s of **D**, H1 of **E** with H2 of **D**, H1 of **B** with H2 of **H**, and H1 of **F** with H2 of **C**. Moreover, the rotating frame Overhauser effect (ROE) contact between H5 of residue **D** and H3ax of **I** suggested a D configuration for Kdo8N [30]. Finally, ROE contacts between H1 of **G** and both H6s of **A** were found.

**Figure 7:**
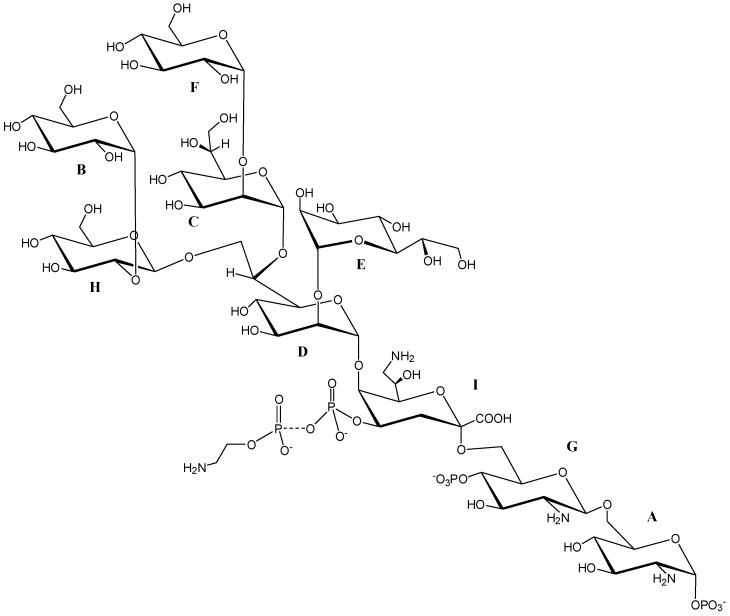
**OS** structure of the LOS from *Shewanella* sp. HM13.

All the reported data allowed us to determine the complete structure of the saccharidic backbone of the *Shewanella* sp. HM13 LOS, which is shown in Figure 7.

## 3. Materials and Methods

### 3.1. Bacteria Growth and LPS Isolation

*Shewanella* sp. HM13, a psychrotrophic bacterium isolated from the intestine of horse mackerel (*Trachurus japonicus*), was cultured in 5 mL LB liquid medium overnight at 18 °C. Five milliliters of the culture were inoculated into 1 L LB liquid medium, and the cells were grown at 4 °C until the OD_600_ reached 3.0–4.0. The cells were harvested at 6800× *g*, 4 °C for 10 min. The cell pellets collected from 2 L cultures were freeze-dried. Dried bacterial cells (1.4 g) were extracted by the PCP method to give 34 mg of LOS (yield 2.4% *w*/*w* of dried cells), and then by the hot phenol/water method as reported previously [19,20].

### 3.2. DOC-PAGE Analysis

Polyacrylamide gel electrophoresis analysis (PAGE) was performed using the system of Laemmli [31] with sodium deoxycholate (DOC) as the detergent, as already described [32]. The gels were fixed in an aqueous solution of 40% ethanol and 5% acetic acid. The LOS extract bands were visualized by silver staining as previously described [33].

### 3.3. Sugar and Fatty Acids Analysis

The LOS sample (0.5 mg) was subjected to a methanolysis reaction with HCl/CH_3_OH (1.25 M, 1 mL) at 80 °C for 16 h. The obtained monosaccharides were acetylated and analyzed as acetylated methyl glycosides by GC-MS. The analysis of the fatty acids, derivatized as methyl esters, was achieved as already reported [34].

The absolute configurations of the glucose and glucosamine were determined by gas chromatography of the acetylated (*S*)-2-octyl glycosides [35]. The heptose configurations were obtained through the alditol acetates GC-MS method. Briefly, a sample of the LOS (0.5 mg), after a methanolysis reaction, was hydrolyzed at 120 °C with 2M trifluoroacetic acid (TFA) for 2 h, as already reported [36]. After neutralization, the sample was reduced with NaBD_4_ and finally acetylated and injected into the GC-MS. The configuration was obtained by comparison with authentic standards.

All the sample derivatives were analyzed on an Agilent Technologies gas chromatograph 6850A equipped with a mass selective detector 5973N and a Zebron ZB-5 capillary column (Phenomenex, Bologna, Italy 30 m × 0.25 mm i.d., flow rate 1 mL/min, He as carrier gas). Acetylated methyl glycosides were analyzed using the following temperature program: 140 °C for 3 min, then 140→240 °C at 3 °C/min. Analysis of acetylated octyl glycosides was performed as follows: 150 °C for 5 min, then 150→300 °C at 6 °C/min, and finally 300 °C for 5 min. The temperature program for methyl esters of fatty acids was the following: 140 °C for 3 min, then 140→280 °C at 10 °C/min, and finally 280 °C for 20 min. The temperature program for alditol acetates was the following: 150 °C for 3 min, and then 150→330 °C at 3 °C/min.

### 3.4. Deacylation of the LOS

The LOS sample (30 mg) was dried over phosphorus anhydride in a vacuum chamber and then treated with hydrazine (1.5 mL) at 37 °C for 2 h. The precipitation of the LOS-OH was obtained by addition of cold acetone. The pellet was recovered after centrifugation (4 °C, 7000 rpm, 30 min), washed two times with cold acetone to remove the excess of hydrazine, suspended in water, and finally freeze-dried [37]. The LOS-OH (8 mg) was submitted to a reaction with KOH 4 M aq. (1.0 mL) for 16 h at 120 °C. The crude reaction was neutralized with HCl 2 M aq. (until pH 6) and extracted with CHCl_3_ for three times. The aqueous phase of the mixture was desalted on a Sephadex G-10 column (GE Healthcare, Pittsburgh, PA, USA, 2.5 × 43 cm, 31 mL h^−1^, fraction volume 2.5 mL, eluent NH_4_HCO_3_ 10 mm). The eluted oligosaccharide fraction was freeze-dried (1.5 mg).

### 3.5. Methylation Analysis

The linkage positions of the monosaccharides were obtained by the analysis of the partially methylated alditol acetates (PMAAs). The methylation reaction was achieved by incubating 1 mg of the LOS sample with CH_3_I (100 µL) and NaOH powder in dimethyl sulfoxide (DMSO, 300 µL) for 20 h [38]. The mixture was analyzed by GC-MS with the following temperature program: 90 °C for 1 min, then 90→140 °C at 25 °C/min, then 140→200 °C at 5 °C/min, then 200→280 °C at 10 °C/min, and finally 280 °C for 10 min.

### 3.6. Mass Spectrometry Analysis

MALDI-TOF mass spectra were acquired on a ABSCIEX TOF/TOF™ 5800 (AB SCIEX, Darmstadt, Germany) mass spectrometer equipped with an Nd:YLF laser with a λ of 345 nm, a < 500-ps pulse length, and a repetition rate of up to 1000 Hz. Approximately 2000 laser shots were accumulated for each spectrum. The calibration of the mass spectra was obtained with a hyaluronan oligosaccharides mixture. A solution of 2,5-dihydroxybenzoic acid (DHB) in 20% CH_3_CN in water (25 mg mL^−1^) was used as the matrix. The samples were desalted on a Dowex 50WX8 (H^+^ form) and dissolved in 2-propanol/water with a 1:1 ratio. The spectra were calibrated and processed under computer control by using the Data Explorer software (v0.2.0).

### 3.7. NMR Spectroscopy

1D and 2D NMR spectra were obtained using a Bruker Avance 600 MHz spectrometer (Billerica, MA, USA) equipped with a cryoprobe. All the NMR experiments (COSY, TOCSY, ROESY, DEPT-HSQC, and HMBC) were performed using standard pulse sequences available in the Bruker software (version 2.1) [39]. NMR spectra were recorded at 298 K, and the mixing time for the TOCSY and ROESY experiments was 100 ms. All the experiments were performed by using sodium 3-trimethylsilyl-(2,2,3,3-^2^H_4_)-propanoate (TSP, δ_H_ 0.00) and 1,4-dioxane in D_2_O (δ_C_ 67.40) as external references. ^31^P and ^1^H-^31^P spectra were recorded at 298 K using a Bruker Ascend 400 MHz spectrometer (Billerica, MA, USA).

## 4. Conclusions

In this paper, the complete structure of the sugar backbone of the LOS from the cold-adapted *Shewanella* sp. HM13 is reported. The structure has been obtained by chemical analysis, NMR spectroscopy, and MALDI-TOF mass spectrometry.

The oligosaccharide shares some structural features with those isolated from other *Shewanella* strains. The presence of the Kdo8N is confirmed as a hallmark of *Shewanella* species, having been already reported for the species *oneidensis* [23], *algae* [27], *putrefaciens* [40], *pacifica* [24], and *Shewanella* spp. MR-4 [21]. In addition, the d,d-configured heptose holds a central position in the oligosaccharide structure, as well as for all the other characterized LOS structures [21,23,24,27,40].

The rough nature of the *Shewanella* sp. HM13 LPS is not surprising, since this has also been reported for other *Shewanella* species [21,23,24,27,40]. Nevertheless, it is worth noting that this feature is common among the LPSs isolated from cold-adapted Gram-negative bacteria [41,42,43,44,45]. A possible explanation lies in the enhanced flexibility and stability of the outer membrane when the O-polysaccharide chain is absent [46].

Finally, to shed light on the possible structural differences among OM components of both bacterial cells and OMVs, it will be very interesting to characterize the LPS isolated from *Shewanella* sp. HM13 OMVs.

## Figures and Tables

**Table 1:** ^1^H and ^13^C assignments of the oligosaccharide (**OS**) obtained from the LOS of *Shewanella* sp. HM13. All the values are referred to sodium 3-trimethylsilyl-(2,2,3,3-^2^H_4_)-propanoate (TSP, δ_H_ 0.00) and 1,4-dioxane in D_2_O (δ_C_ 67.40) as external standards. Spectra were recorded at 298 K at 600 MHz.

Residue	H1 C1 ^1^*J*_C,H_	H2 C2	H3 C3	H4 C4	H5 C5	H6a C6	H6b	H7a C7	H7b	H8a C8	H8b
**A** α-GlcN*p1P*	5.65 92.0 179	3.36 55.8	3.91 71.2	3.46 71.4	4.20 73.5	3.91 70.9	4.32				
**B** α-*t*-Glc*p*	5.40 99.1 182	3.58 72.5	3.76 74.0	3.49 70.7	4.04 73.5	3.76 61.9					
**C** α-2-l,d-Hep*p*	5.37 98.8 184	3.91 82.1	4.05 71.7	3.83 68.7	3.91 75.2	4.07 70.3		3.78 64.7	3.75		
**D** α-2,6,7-d,d-Hep*p*	5.17 101.9 170	4.08 79.4	4.15 71.7	3.79 68.7	4.06 74.4	4.34 78.2		4.02 70.8	4.13		
**E** α-*t*-l,d-Hep*p*	5.12 103.5 173	4.07 70.5	3.83 72.0	3.86 67.6	3.66 73.8	4.06 70.5		3.84 63.5	3.72		
**F** α-*t*-Glc*p*	5.05 102.3 170	3.59 73.1	3.72 74.2	3.49 70.8	4.04 73.0	3.81 61.8					
**G** β-6-GlcN*p4P*	4.95 100.8 164	3.11 56.9	3.80 73.6	3.85 74.5	3.75 75.4	3.51 63.4	3.73				
**H** β-2-Glc*p*	4.65 105.0 164	3.47 77.1	3.57 75.9	3.42 71.4	3.44 78.5	3.73 62.3	3.93				
**I** α-5-Kdo8N*p4P*	n.d.	101.2	2.01, 2.25 35.8	4.58 71.3	4.34 76.3	3.92 74.6		4.02 67.0		3.20 44.4	3.50
